# Performance of High Layer Thickness in Selective Laser Melting of Ti6Al4V

**DOI:** 10.3390/ma9120975

**Published:** 2016-12-01

**Authors:** Xuezhi Shi, Shuyuan Ma, Changmeng Liu, Cheng Chen, Qianru Wu, Xianping Chen, Jiping Lu

**Affiliations:** 1 School of Mechanical Engineering, Beijing Institute of Technology, Beijing 100081, China; shixuezhisheo@gmail.com (X.S.); bitmc@bit.edu.cn (S.M.); cc.lime@hotmail.com (C.C.); qrwu@foxmail.com (Q.W.); jipinglu@bit.edu.cn (J.L.); 2 Beijing Institute of Astronautical Systems Engineering, Beijing 100076, China; chenxianping2000@gmail.com

**Keywords:** selective laser melting, high layer thickness, coarse powders, Ti6Al4V, microstructure, mechanical properties, defects

## Abstract

To increase building rate and save cost, the selective laser melting (SLM) of Ti6Al4V with a high layer thickness (200 μm) and low cost coarse powders (53 μm–106 μm) at a laser power of 400 W is investigated in this preliminary study. A relatively large laser beam with a diameter of 200 μm is utilized to produce a stable melt pool at high layer thickness, and the appropriate scanning track, which has a smooth surface with a shallow contact angle, can be obtained at the scanning speeds from 40 mm/s to 80 mm/s. By adjusting the hatch spacings, the density of multi-layer samples can be up to 99.99%, which is much higher than that achieved in previous studies about high layer thickness selective laser melting. Meanwhile, the building rate can be up to 7.2 mm^3^/s, which is about 2 times–9 times that of the commercial equipment. Besides, two kinds of defects are observed: the large un-melted defects and the small spherical micropores. The formation of the un-melted defects is mainly attributed to the inappropriate overlap rates and the unstable scanning tracks, which can be eliminated by adjusting the processing parameters. Nevertheless, the micropores cannot be completely eliminated. It is worth noting that the high layer thickness plays a key role on surface roughness rather than tensile properties during the SLM process. Although a sample with a relatively coarse surface is generated, the average values of yield strength, ultimate tensile strength, and elongation are 1050 MPa, 1140 MPa, and 7.03%, respectively, which are not obviously different than those with the thin layer thickness used in previous research; this is due to the similar metallurgical bonding and microstructure.

## 1. Introduction

Selective laser melting (SLM) is one of the additive manufacturing techniques in which functional, complex parts are formed by selectively melting successive layers of powder particle using a laser beam. The SLM is the most promising metal additive manufacturing technology that can be widely used in industry, because it can generate metal parts with fine surface roughness, high density, high mechanical properties, and even arbitrary complex structures [1,2,3,4]. However, the applications of SLM technology in industry are still limited, currently, due to its low efficiency and high cost, which are intensely associated with layer thickness and powders.

Generally, during the SLM processing, the layer thickness is very thin, about 20 μm–50 μm [5,6,7,8,9], which results in the high surface precision. However, the thin layers largely restrict the building rate. For some metal parts of which the surface finish requirements are relatively low, high layer thickness can be applied to increase the building rate. Ma et al. investigated the selective laser melting 1Cr18Ni9Ti alloy with the layer thickness from 60 μm to 150 μm, the building rate increased by 10 times–20 times in comparison with that of traditional SLM processing [10]. When Sebastian et al. increased the layer thickness to 200 μm for the production of AlSi10Mg parts, the process-related building rate could be increased from 4 mm^3^/s to 21 mm^3^/s, which equals a 525% growth [11]. The previous studies demonstrated that high layer thickness is an effective way to improve SLM process efficiency.

Besides, high layer thickness can also greatly reduce the cost. As we know, the powder size is limited by the layer thickness. For a thin layer (20 μm–50 μm), the powders used in SLM are very fine (10 μm–50 μm), which are very expensive compared with the traditional metal wire or plate. However, for high layer thickness (>100 μm), the relatively coarse powders with the size range of about 53 μm–106 μm can be used, which are much cheaper than the fine powders due to their high formation rate in the process of fabricating powders. Taking Ti6Al4V as an example, the price of the coarse powder (53 μm–106 μm) is only about 30%–50% of fine powder (10 μm–50 μm). In this case, high layer thickness combined with coarse powder can be used to fabricate parts when the surface finish requirements are relatively low, and this leads to huge cost and time reductions. According to our knowledge, few studies have been carried out on the investigation of SLM with high layer thickness and coarse powders.

Hence, in this paper, the SLM with high layer thickness (200 μm) and coarse powders (53 μm–106 μm) are investigated. The SLM processing is systematically investigated to obtain high density, which is necessary for high mechanical properties. The effects of process parameters on the density, microstructure, and mechanical properties are presented. Meanwhile, the variations in defect morphology with different process parameters and the formation mechanisms of defects are revealed. 

## 2. Experimental Procedures

### 2.1. Materials

**Figure 1:**
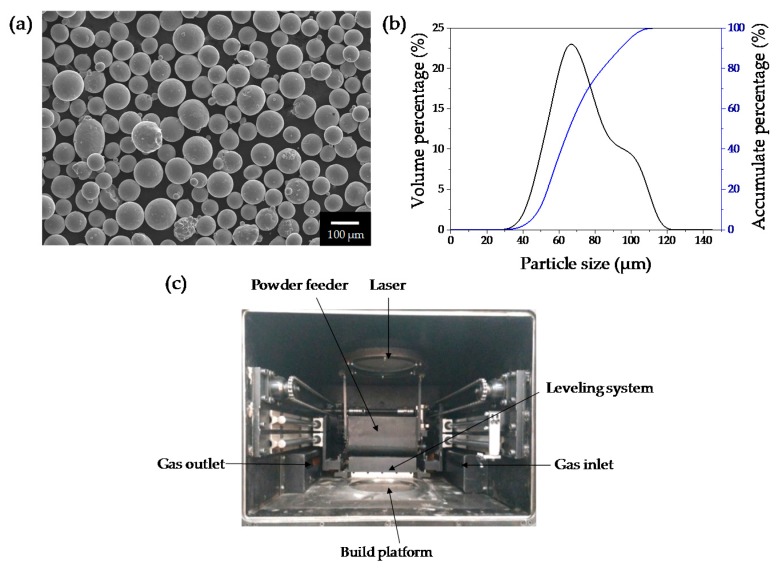
(**a**) Ti6Al4V powder morphology; (**b**) particle size distribution; (**c**) selective laser melting system developed by Beijing Institute of Technology.

The gas atomised Ti6Al4V powder with a size range of 53 μm–106 μm supplied by AVIC BIAM was used in this study. The morphology of powders is shown in Figure 1a. The powder has an apparent density of 2.48 g/cm^3^. Figure 1b shows a distribution with an average particle size of 68 μm (d_10_: 57 μm, d_90_: 95 μm).

### 2.2. Experimental Setup and Manufacturing Process

**Figure 2:**
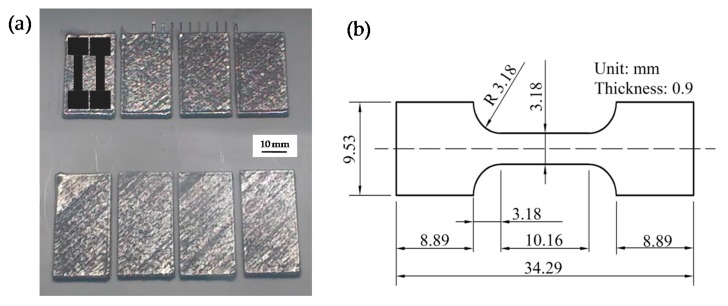
(**a**) The macrograph of the Ti6Al4V samples; (**b**) geometrical shape and size of tensile test specimen.

The SLM equipment utilized to produce Ti6Al4V specimens was developed by the Beijing Institute of Technology (Beijing, China), as shown in Figure 1c. The system uses a fiber laser (YLR-WC, IPG Photonics Corporation, Burbach, Germany) with a maximum power of 500 W in continuous laser mode and a wavelength of 1070 nm. In all the experiments, the laser beam was focused exactly on the substrate surface with a spot size of 200 µm. A commercial Ti6Al4V alloy plate of 10 mm thickness was used as the substrate. The working chamber provided a closed environment which was filled with argon as a protective gas to maintain an oxygen concentration below 100 ppm. In order to identify a range of suitable parameters for manufacturing Ti6Al4V alloy, a series of single tracks with a length of 10 mm were first melted. In order to prevent uneven layer thickness, two tracks were melted at different positions of the substrate in each condition. Single tracks were melted at a constant high laser power (*P* = 400 W) and layer thickness (*δ* = 200 µm), but at different laser scanning speeds ranging from mm/s 40 to 200 mm/s with a step of 20 mm/s. Then, block samples with dimensions of 40 × 20 × 3 mm^3^ (15 layers) were produced (Figure 2a) using various scanning speeds and hatch spacings with a cross scanning strategy, as shown in Table 1.

### 2.3. Characterization

The surfaces of single tracks and the block samples were characterized using a scanning electron microscope (SEM) (JSM6490, JEOL, Tokyo, Japan) after fabrication. After evaluating the surface morphology, all of the single tracks and cubic samples were sectioned by wire-cut machine (Baojun, Suzhou, China), then ground and polished following standard metallographic procedures. Metallographic specimens were prepared by standard mechanical polishing and etched with a solution of 2 mL HF, 6 mL HNO_3_, and 90 mL H_2_O. The cross-sectional microstructures were observed using an optical microscope (OM) (DM4000M, Leica, Wetzlar, Germany) and an SEM. The geometrical characteristics of the single tracks and the density of SLM samples were examined by analyzing optical micrographs and quantified using ImageJ software. For each track and sample, two cross sections at different locations were measured and averaged. The tensile test pieces were machined (see Figure 2a) and examined using an Instron 5966 (Instron, Boston, MA, USA) testing machine to evaluate the tensile properties at room temperature. The displacement rate of cross head was 0.01 mm/s, and a dynamic strain gauge extensometer was applied to record the strain. The tensile properties were calculated by mathematically averaging the test results of at least three tensile samples. The dimensions of tensile specimens are shown in Figure 2b.

## 3. Results and Discussion

### 3.1. Single Scan Tracks

The properties of the parts manufactured by SLM depend strongly on the properties of each single track. By evaluating the scan track characteristics, such as surface morphology and geometric features, significant information on the selection of process parameters can be gained, such as melt pool shape, stability, and wetting. According to the previous studies, to obtain the samples with high density, the proper single scanning track should be continuous and smooth with a shallow contact angle [12,13,14,15]. Hence, in this section, the surface morphology and geometric characteristics of the single scanning tracks are investigated.

#### 3.1.1. Surface Morphologies

**Figure 3:**
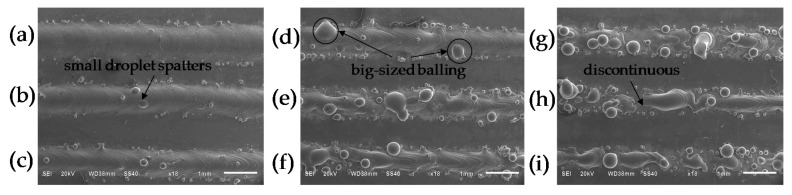
SEM micrographs showing the surface of single scan tracks under different scanning speeds (*P* = 400 W, *δ* = 200 μm). (**a**) *v* = 40 mm/s; (**b**) *v* = 60 mm/s; (**c**) *v* = 80 mm/s; (**d**) *v* = 100 mm/s; (**e**) *v* = 120 mm/s; (**f**) *v* = 140 mm/s; (**g**) *v* = 160 mm/s; (**h**) *v* = 180 mm/s; (**i**) *v* = 200 mm/s.

Figure 3 depicts the surface morphology of single scan tracks under different scanning speeds. It can be seen that as the scanning speed increases, the scan track surfaces change from smooth to rough. Meanwhile, the scan tracks become discontinuous and break up into balling. At lower scanning speeds from 40 mm/s to 80 mm/s, the scan tracks are continuous and give rise to a smooth surface with only small-sized droplets beside the “tracks” as shown in Figure 3a–c. As the scanning speed is increased to 140 mm/s, the scan tracks are inclined to be discontinuous, leading to a relatively coarse surface attached with large balling (see Figure 3d–f). With the further increase of scanning speed to 200 mm/s, the balling effect is intensified sequentially, and the scan tracks are completely discontinuous (see Figure 3g–i).

The observations suggest that the melt pool will become increasingly unstable with the increased scanning speed; the reasons are as follows: During the SLM process, the dynamic viscosity of a melt pool with an entirely liquid formation is temperature-dependent [16]. Using a lower scanning speed of 40 mm/s leads to longer dwelling time of the laser on the surface of melt pool, with attendant higher laser energy input to the pool. The operative temperature of the Ti6Al4V liquid within the pool accordingly increases, which gives rise to a larger amount of liquid formation with lower viscosity. The combined influence of a long liquid lifetime and low viscosity results in a high degree of overheating of the Ti6Al4V liquid and the attendant elevated melt pool instability. Therefore, a number of small-sized liquid droplets splash from the liquid front being solidified [7,16]. At a relatively high scanning speed of 100 mm/s, the energy input is insufficient to achieve complete melting of the powder and substrate material, a smaller melt pool is created, and when its length exceeds its circumference, the melt pool easily breaks into balls as a result of Rayleigh instability [14]. At an even higher scanning speed of 160 mm/s, the energy density is too low to fully melt the powder and substrate, and a number of balls occur and result in the formation of discontinuous scan tracks. In addition, a high temperature gradient forms during the SLM process, leading to convective movement of the molten pool and the resultant shear stress as well as a surface tension gradient [17]. When the scan speed decreases, more heat input is injected into the molten pool and then greater shear stress is obtained, which can also cause instability of the melt pool. 

#### 3.1.2. Geometrical Characteristics

**Figure 4:**
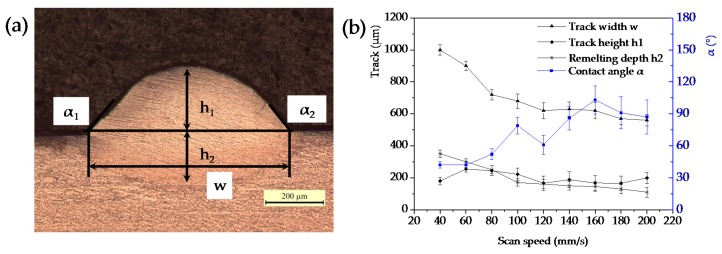
(**a**) Measurement of the cross section of the scan track geometry; (**b**) the geometrical characteristics of synthesized single tracks as a function of scanning speed.

Obviously, the scanning speed has a statistically significant influence on the behavior of individual tracks and their geometric characteristics. Furthermore, in order to better analyze and quantify the effect of scanning speed on the geometrical characteristics of the single tracks, the cross section of each track was studied. Geometrical characteristics of the cross sections of single tracks were measured as shown in Figure 4a, including track width (*w*), height (*h*_1_), remelting depth (*h*_2_), and average value of contact angle (*α* = (*α*_1_ + *α*_2_)/2). Figure 4b shows the relationship between the geometrical characteristics of synthesized single tracks and the scanning speeds.

As shown in Figure 4b, with the increase of scanning speed, the track width and remelted depth decrease from 1000 μm to 560 μm and from 350 μm to 110 μm, respectively. The track height fluctuates in the value of 200 μm, but the depth of melt pool (track height + remelted depth) reflects a downward trend, decreasing from 530 μm to 300 μm as the scanning speed increases and maintains a comparatively stable value of 300 μm at higher speeds from 120 mm/s to 200 mm/s. The contact angle shows a trend of increase with the increase of scanning speed, the steep angle is greater than 60° when the scanning speed is more than 100 mm/s. Therefore, the track shape is larger and deeper at slower scanning speed, and the melt pool inclines to curl into a ball at a high scanning speed.

**Figure 5:**
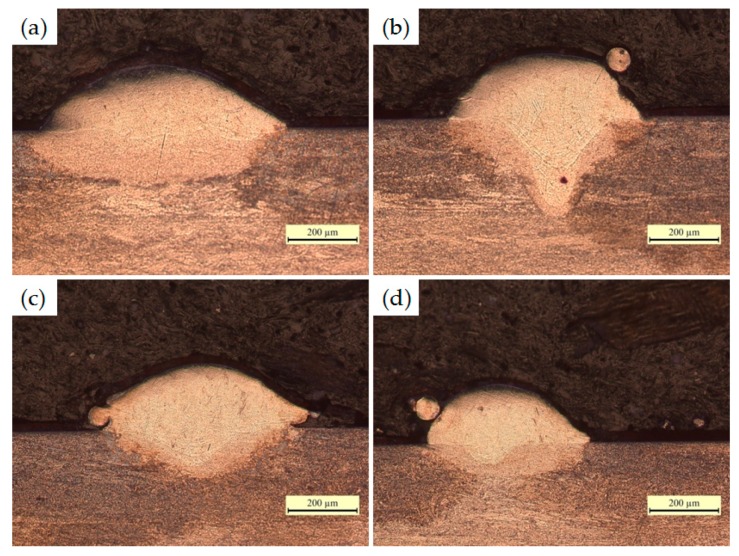
OM micrographs exposed on the cross section of single scan tracks as the scanning speed increased (*P* = 400 W, *δ* = 200 μm). (**a**) *v* = 80 mm/s; (**b**) *v* = 120 mm/s; (**c**) *v* = 160 mm/s; (**d**) *v* = 200 mm/s.

With the increase of scanning speed, the melt pool leads to unfavorable wetting, flowing, spreading characteristics, and corresponding balling phenomenon, as shown in Figure 5. The low remelted depth and steep contact angle when the scanning speeds are higher than 100 mm/s suggest the wetting behavior between melt pool and substrate becomes worse. Thus, combined with the content of the surface morphology and the cross sectional characteristic of single scan tracks, these process parameters (high scanning speeds (*v* > 100 mm/s)) are unsuitable for powder bed melt processes, and the stable melt pools (width (1000 μm–720 μm), height (180 μm–244 μm), remelted depth (350 μm–250 μm), and contact angle (42°–52°)) are obtained at the scanning speeds between 40 and 80 mm/s combined with power of 400 W and layer thickness of 200 μm for a successful SLM process.

### 3.2. Multi-Layer Fabrication

#### 3.2.1. Density Behavior

Based on the above investigations, the scanning speeds of 40, 60, and 80 mm/s were selected to be applied to fabricate multi-layer samples, meanwhile, 100 mm/s was chosen as a comparison group. The samples were produced using various scanning speeds and hatch spacings as shown in Table 1, the hatch spacing was varied based on the width *w* of the scan track (Figure 4b). The effect of hatch spacings on density of SLM Ti6Al4V was studied.

**Figure 6:**
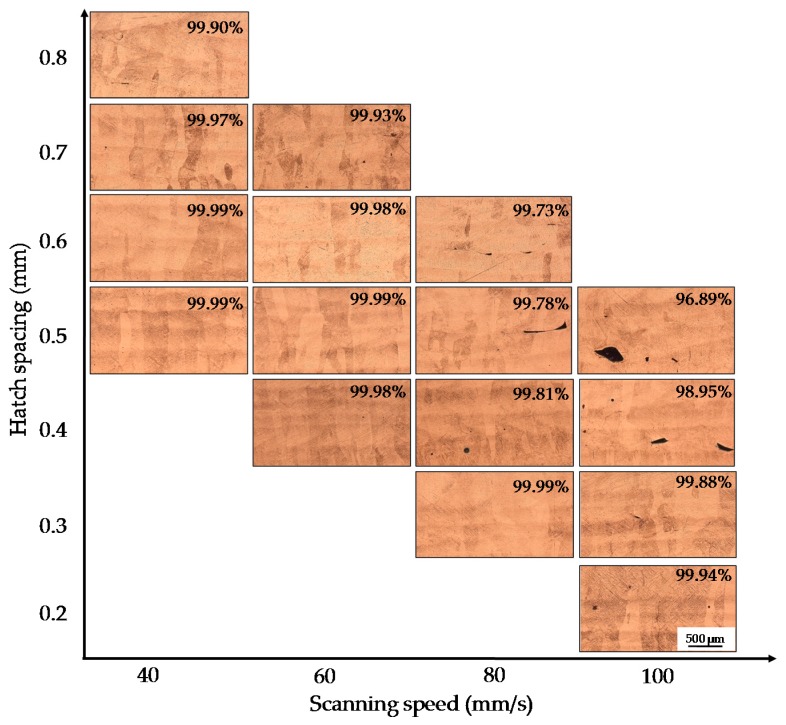
Density behaviors of selective laser melting Ti6Al4V samples with different scanning speeds and hatch spacings (*P* = 400 W, *δ* = 200 μm).

Figure 6 shows the density behaviors of the samples with different scanning speeds and hatch spacings. Firstly, we found that the SLM processing with high layer thickness and coarse powders can obtain high density, which can be up to 99.99%. Meanwhile, it can be seen that the scanning speed and hatch spacing largely influence the density. For example, when the hatch spacing was fixed at 0.5 mm, the density droped significantly from 99.99% to 96.89% as the scanning speed increased from 40 mm/s to 100 mm/s. When the scanning speed was fixed at 80 mm/s, the density droped from 99.99% to 99.73% as the hatch spacing increased from 0.3 mm to 0.6 mm. Besides, with the increase of scanning speed, the variation of hatch spacings had a more intense impact on the density. At the speeds of 40 and 60 mm/s, the variation of hatch spacings had no dramatic effects on density, and the densities declined slightly from 99.99% to 99.90% and from 99.99% to 99.93%, respectively. At the speed of 80 mm/s, the density had a larger fluctuation from 99.99% to 99.73% with increasing hatch spacing from 0.3 mm to 0.6 mm. At the speed of 100 mm/s, the density decreased sharply from 99.94% to 96.89% with increasing hatch spacing from 0.2 mm to 0.5 mm.

The above results suggest that high density (99.99%) can be easily obtained under the relatively large range of SLM processing parameters in this study. However, in the previous studies, when the layer thickness is high, the samples generally are very porous and exhibit low density [10,18,19,20]. The reasons are explained as follows.

**Figure 7:**
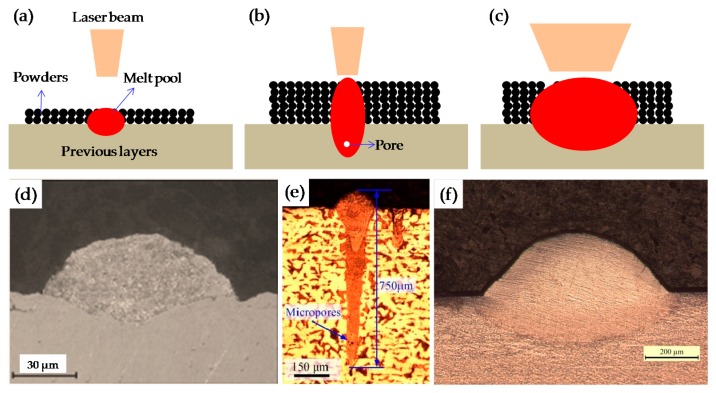
(**a**–**c**) Schematic sketches of the selective laser melting (SLM) process of different melt pools; (**a**) fabrication at a thin layer with a small-diameter laser; (**b**) fabrication at a thick layer with a small-diameter laser; (**c**) fabrication at a thick layer with a large-diameter laser; (**d**–**f**) OM micrographs of the cross section of single scan tracks at different layer thicknesses; (**d**) *δ* = 50 μm [13]; (**e**) *δ* = 100 μm [10]; (**f**) *δ* = 200 μm.

Generally, the SLM equipment used mostly had lasers with spot sizes of 50 µm–100 µm in order to guarantee a stable melt pool (see Figure 7a) and obtain high relative density metal parts at thin layer thickness. Yadroitsev et al. successfully fabricated the maximum density of the SLM samples (less than 1% porosity) with the layer thickness of 50 µm and the laser spot diameter of 70 µm (see Figure 7d) [13]. However, at a thicker layer thickness, the higher energy density is needed to melt the thicker powder layer; however, increasing the laser power and maintaining a small beam diameter (50 µm–100 µm) leads to process instabilities [21]. Excessively high laser energy density easily leads to improper closure of the keyhole, as shown in Figure 7b, which may be caused by entrapped gases and a higher evaporation rate under a higher laser energy input [22,23]. Ma et al. found that at the thicker layer thickness of 100 μm, the residual gas at the bottom of the larger melt pools cannot escape in time during rapid solidification and, thus, forms pores in the fabricated samples, as indicated by the blue single-head arrow in Figure 7e [10]. To avoid these process instabilities, the laser beam diameter has to be enlarged with an increase of laser power. Schleifenbaum et al. achieved satisfactory results for SLM metallic powders by means of increased laser power (up to 500 W) and the correspondent adaption of the beam diameter to approximately 0.8 mm [21]. Sebastian et al. increased the layer power to be 1000 W with a beam diameter of 1000 µm for the production of AlSi10Mg parts, and a density approaching 100% without cracks and fusion defects could be obtained [11]. In this study, in order to obtain stable melt pool and full-density samples at the high layer thickness of 200 µm, the laser spot diameter was expanded to 200 µm, in this way, the larger laser beam could produce a larger and shallower melt pool (Figure 7c) that ensured the powder was fully melted and did not produce a deeper bonding area as expected (Figure 7f).

#### 3.2.2. Building Rate

The building rate is directly proportional to the parameters of layer thickness (*δ*), hatch spacing (*s*), and scanning speed (*v*). The layer thickness and scanning speed are limited by the available laser power. The hatch spacing is limited by the diameter of the beam and typically equals approximately 0.7 times the beam diameter [11]. A benchmark to measure the productivity of the SLM process is given by the process-related building rate, which is determined by the product of layer thickness, hatch spacing, and scanning speed according to the equation:

*V_b_* = *δ* × *s* × *v* (1)

where *V_b_* is the building rate (mm^3^/s). Therefore, the most efficient method to increase the building rate is matching a thicker powder layer with a higher scanning velocity under larger hatch spacing [21]. In our experiments, the stable melt pool had a larger width ranging from 1000 μm to 720 μm, so the proper hatch spacing was approximately 600 μm, which largely increased the building rate. The fast building rate reached to 4 mm^3^/s–7.2 mm^3^/s, while a density of at least 99.98% was achieved (*P* = 400 W, *δ* = 200 µm, *v* = 60 mm/s, *s* = 600 µm, maximum building rate of 7.2 mm^3^/s). In previous studies, SLM machines were equipped with laser power up to 200 W (max = 400 W) and a focus diameter of approximately 100 µm, so building rates for the production of Ti6Al4V parts ranged from 0.5 mm^3^/s to 4.5 mm^3^/s. Table 2 exhibits a comparison of building rates between the present study and the previous research, for example, M. Simonelli et al. [24] and Gong et al. [19] separately use AM250 (Renishaw, London, UK) and EOS M270 (Gmbh, Planegg, Germany) to fabricate Ti6Al4V samples, the building rate of which is 1.135 mm^3^/s and 2.4 mm^3^/s, respectively. Therefore, by increasing the layer thickness to 200 μm for the production of Ti6Al4V parts, the building rate can reach 7.2 mm^3^/s, which is about 2 times–9 times that of the commercial equipment.

#### 3.2.3. Mechanisms of Defects Forming

**Figure 8:**
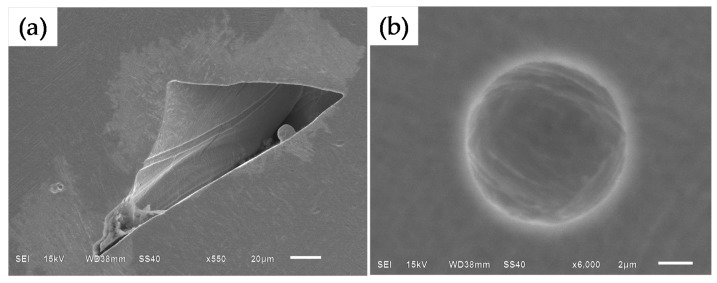
SEM micrographs show the two different defects morphologies; (**a**) large irregular defects; (**b**) small spherical micropore. (*P* = 400 W, *δ* = 200 μm).

**Figure 9:**
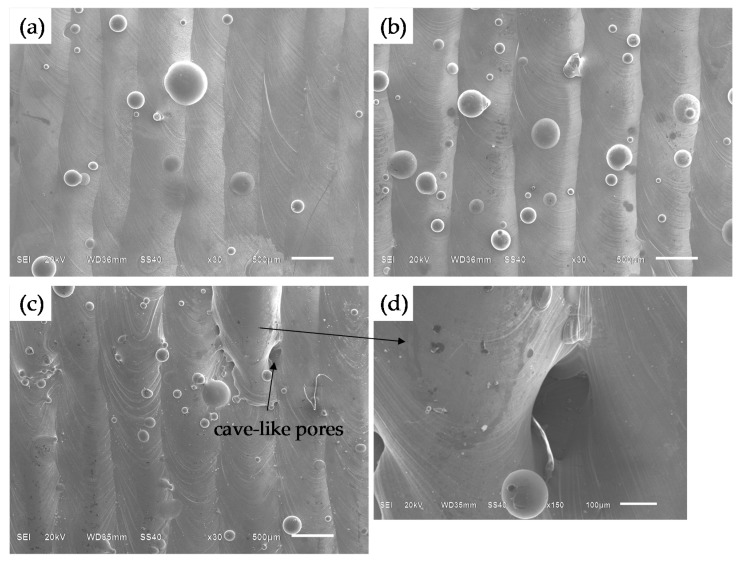
SEM micrographs show the top surface structure of the samples (*P* = 400 W, *δ* = 200 μm): (**a**) *v* = 40 mm/s, *s* = 0.5 mm; (**b**) *v* = 40 mm/s, *s* = 0.8 mm; (**c**,**d**) *v* = 100 mm/s, *s* = 0.5 mm.

**Figure 10:**
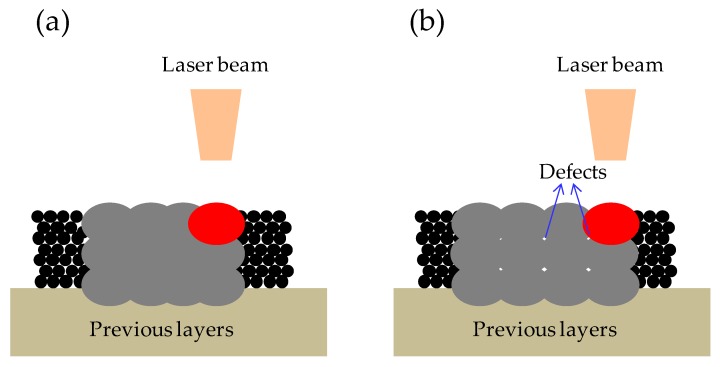
Schematic sketch of SLM process showing the formation of defects; (**a**) small hatch spacing; (**b**) large hatch spacing.

The density of all the samples in this study ranged from 99.65% to 99.99% (see Figure 6). Meanwhile, it can be found that there were two different defect morphologies in the specimens’ interior: the small spherical micropores and the large irregular defects, as shown in Figure 8. To understand the formation mechanisms of defects, cross sections of the samples were investigated. It is noted that at lower scanning speeds from 40 mm/s to 80 mm/s the irregular-shaped defects (Figure 8a), generally below 1000 μm^2^, only occur at large hatch spacings when the overlap rate is less than 30%. This is because the hatch spacing will influence the overlap between two adjacent scan tracks, and inappropriate overlap rate will deteriorate the top surface roughness and produce many rugged hollows due to the cumulative effect [27]. At scanning speeds between 40 and 80 mm/s, the tracks were continuous and regular (Figure 3a–c), when the overlap rate was up to 50% at small hatch spacing, the tracks were evenly lined up and homogeneously overlapped with neighboring tracks (Figure 9a), whereas at the large hatch spacing of 0.8 mm with an overlap rate of 20%, the top surface roughness deteriorated and more balling particles were observed (Figure 9b). Therefore, large hatch spacing leads to a rough surface which may impede the melted liquid from fillling the hollow in the previous solidified layer; this results in the formation of defects. In this case, the schematic sketch of the formation of defects is illustrated in Figure 10. It can be seen that small hatch spacing had a high overlap rate which was more likely to fill the hollow in the previous solidified layer for no-defect interface formation (Figure 10a). More and larger un-melted defects mainly occur at large hatch spacing (Figure 10b) due to the small overlap rate.

At an even higher speed of 100 mm/s, the larger defects were found to be dispersed within interlayer bonding areas on the cross-section of the SLM part. These defects became larger with an average area of 1000 to 30,000 μm^2^ with the increase of hatch spacing. The formation of defects is ascribed to the occurrence of the balling phenomenon (Figure 3d) [28] and the cave-like pores that formed on the top surface (Figure 9c,d) [29]. Therefore, porosity is visible in all samples with different hatch spacings at the speed of 100 mm/s (Figure 6). The result also shows that the surface morphology is related to the formation of defects, which is strongly associated with the properties of the single scanning tracks, and it is difficult to achieve high density samples through stacking unstable scanning tracks.

However, the relative density cannot reach 100% in spite of using different process parameters due to the residual micropores. These spherical pores are usually very small (approximate 15 µm, Figure 8b). The study found that micropores exist in random locations within the specimens, and the formation of the spherical pores has no clear relationship with the scanning speed and hatch spacing. Whether at the low speed of 40 mm/s and large hatch spacing of 0.6 mm or at the high speed of 100 mm/s and small hatch spacing of 0.2 mm, the internal micropores could be formed. The main reason is that the rapid solidification rate is higher than the buoyancy velocity of the entrapped gas in the melting process. When a laser spot with high energy density is applied to a powder bed, gas bubbles are entrapped in the melt pool because materials evaporate and the residual gas cannot escape in time [30,31]. It has also been found that the micropores may originate from hollow gas-atomized powder and remain in as-built material [27,32,33]. Therefore, the formation of internal micropores is not only related to the energy input, but also depends on the characteristics of the powder. It is unlikely that optimizing the process parameters would eliminate completely these micropores, so a full dense sample cannot be obtained.

#### 3.2.4. Microstructure and Tensile Properties

**Figure 11:**
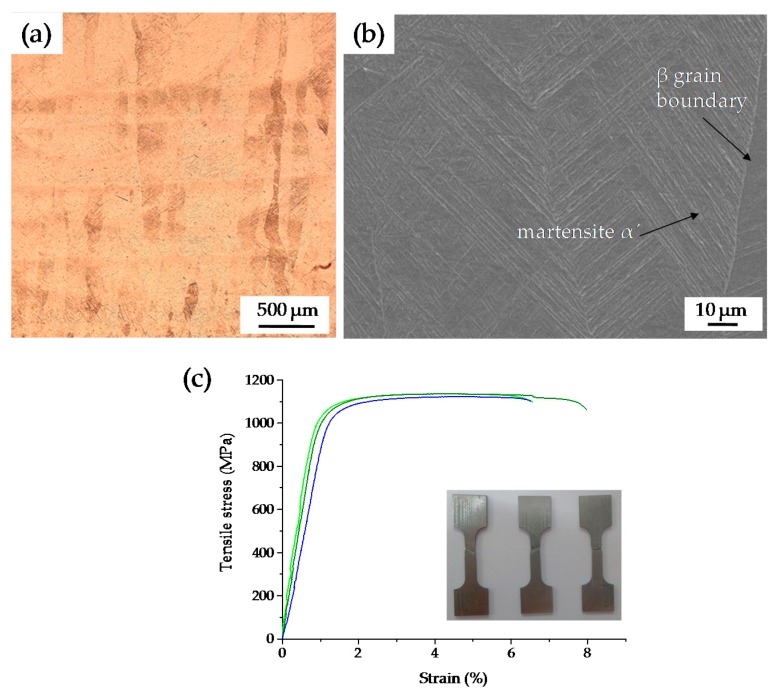
The sample with greatest density and fastest building rate was fabricated at a scanning speed of 60 mm/s and hatch spacing of 0.5 mm. (**a**) optical micrograph of columnar prior-*β* grains; (**b**) SEM image of the typical martensite microstructure; (**c**) Stress-strain plots of the sample.

Figure 11 shows a vertical section of the microstructure of the SLM sample that was fabricated at the scanning speed of 60 mm/s and hatch spacing of 0.5 mm. It illustrates that the microstructure of Ti6Al4V consisted of a mixture of *α* and *β* phases. Figure 11a shows the coarse *β* columnar grains grew along the building direction of the sample due to a substantial thermal gradient that existed along the building direction in the SLM process. The grain width ranged from 40 μm to 150 μm, and the average grain size was 80 μm. A comparison of the microstructure of SLM Ti6Al4V at a thin layer thickness of 30 μm by Thijs et al. [25] led to a similar result; they obtained coarse grains with the average width between 50 and 100 μm, and Vrancken et al. also got similar results [34]. The morphology of acicular martensite *α*´ can be observed from the SEM-SE image shown in Figure 11b. Majorities of acicular martensite originate from the prior *β* grain boundaries and fill the columnar grains. This is attributed to the high cooling rate in the SLM process, which is more than the critical cooling rate of 410 °C/s that allows the formation of a full martensite of Ti6Al4V [35]. 

As shown in Figure 11c, the three SLM specimens have stable values of yield strength, ultimate tensile strength, and elongation. Ultimate tensile strength ranges from 1130 MPa to 1145 MPa, yield strength ranges from 1040 MPa to 1060 MPa, and elongation ranges from 6.52% to 7.99%. The average values of yield strength, ultimate tensile strength, and elongation are 1050 MPa, 1140 MPa, and 7.03%, respectively. A comparison of tensile properties between the high layer thickness and the thin layer thickness is listed in Table 3. It indicates that good mechanical properties can be obtained in high layer thickness of 200 μm, and there are no obvious differences between the high layer thickness and thin layer thickness due to the similar metallurgical bonding and microstructure.

It is worth noting that the high layer thickness plays a key role on surface roughness rather than tensile properties during SLM process [38]. In our experiments, a large number of small-sized droplets were spattering seriously during the SLM process and the sample with a relative coarse surface was generated, as shown in Figure 2a and Figure 9. This occurs mainly because high layer thickness needs high laser power and high energy input to fully melt the powder; however, the intensity of the laser energy is increased whereby the evaporation rate rises and a higher incidence of spattering occurs [11,21]. Meanwhile, high laser power offers huge recoil pressure acting on the surface of the overheated liquid which causes the sample surface to become uneven. This behavior warrants further study to improve the surface precision in a subsequent experiment. 

## 4. Conclusions

In summary, selective laser melting technology has been used to build Ti6Al4V samples by melting coarse powder ranging from 53 μm to 106 μm in a thicker powder layer of 200 μm. The main findings are as follows:

(1) The series of single track experiments showed that a stable and proper melt pool can be obtained at lower scanning speeds from 40 mm/s to 80 mm/s combined with a laser power of 400 W and a laser beam diameter of 200 μm.
(2) All the fabricated samples were very close to full density ranging from 99.73% to 99.99%. The building rate was up to 7.2 mm^3^/s, which is about 2 times–9 times that of the commercial equipment.
(3) There are two different defect morphologies in the specimens’ interior, the large un-melted defects and the small spherical micropores. The formation of the un-melted defects was mainly attributed to the inappropriate overlap rates and the unstable scanning tracks, which can be eliminated by adjusting the processing parameters. Nevertheless, the micropores cannot be completely eliminated.
(4) The microstructure of SLM samples consisted of a mixture of *α* and *β* phases. The coarse *β* columnar grains grew along the building direction of the SLM samples. The majority of acicular martensite *α*′ originate from the prior *β* grain boundaries and filled the columnar grains.
(5) The high layer thickness played a key role on surface roughness rather than tensile properties during the SLM process. Although a sample with a relatively coarse surface was generated, the average values of yield strength, ultimate tensile strength, and elongation were 1050 MPa, 1140 MPa, and 7.03%, respectively, which are not obviously different than those of the thin layer thickness in the previous research; this is due to the similar metallurgical bonding and microstructure.

## Figures and Tables

**Table 1:** The process parameters for the different samples.

Scanning Speed *v* (mm/s)	Hatch Spacing *s* (mm)			
40	0.5	0.6	0.7	0.8
60	0.4	0.5	0.6	0.7
80	0.3	0.4	0.5	0.6
100	0.2	0.3	0.4	0.5

**Table 2:** Summary of building rates with regard to the SLM of Ti6Al4V.

Particle Size (µm)	Layer Thickness (µm)	Hatch Spacing (µm)	Scanning Speed (mm/s)	Building Rate (mm^3^/s)	References
d_50_ = 32	50	150	600	4.5	[4]
d_50_ = 35	50	40	400	0.8	[7]
d_50_ = 38	30	100	800	2.4	[19]
d_50_ = 30	30	100	540	2.88	[22]
d_50_ < 50	50	100	227	1.135	[24]
d_50_ = 34	30	75	200	0.45	[25]
d_50_ = 35	30	200	500	3	[26]
d_50_ = 68	200	600	60	7.2	In this study

**Table 3:** Tensile properties of SLM samples at thick layer thickness (200 μm) and thin layer thicknesses (0 μm–50 μm).

Layer Thickness (μm)	Yield Stress (Offset 0.2%) (MPa)	Ultimate Tensile Stress (MPa)	Elongation (%)	References
<50	1333	1407	4.54	[3]
<50	1195	1269	5	[5]
<50	1098	1237	8.8	[22]
30	1110	1267	7.28	[34]
30	1140	1214	3.2	[36]
<50	990	1095	8.1	[37]
200	1050	1140	7.03	In this study
